# Metabolic Reponses to a physical exercise session in women with excess body mass: randomized clinical trial

**DOI:** 10.1186/s12944-017-0600-9

**Published:** 2017-12-19

**Authors:** Djeyne Silveira Wagmacker, Jefferson Petto, Amanda Silva Fraga, Jackeline Barbosa Matias, Sindy Kerole Andrade Mota, Luiz Erlon Araujo Rodrigues, Ana Marice Ladeia

**Affiliations:** 1 College Adventist Bahia, Cachoeira, Bahia Brazil; 2 Bahian School of Medicine and Public Health, Science Development Foundation of Bahia, Bahia, Brazil; 3 Catholic University of Salvador, Salvador, Bahia Brazil; 4 Present Address: Br 101, Km 197, cx postal 18, Salvador, Bahia Brazil

**Keywords:** Obesity, Motor activity, Lipids, Glycemia

## Abstract

**Background:**

There are various factors that influence the effect of physical exercise on the lipid profile, among them the body mass index and calorie expenditure of the exercise are some of the main factors. To test the hypothesis that a physical exercise session based on caloric expenditure may acutely modify the glycemia and lipid values of women with excess body mass.

**Methods:**

The study included 66 women, randomly divided into two groups, control and experimental, with BMI of 29 ± 4.4 kg/m^2^ vs 29 ± 4.3 kg/m^2^ (*p* = 0.45) sedentary and aged 23 ± 3.8 vs 24 ± 3.5 years, respectively (*p* = 0.25). After 12 h fasting, the volunteers underwent the first blood collection. The experimental group was submitted to a physical exercise session corresponding to energy expenditure of 250Kcal, of light intensity based the Borg Rating of Perceived Exertion (RPE), 12 h after the first blood collection. The control and experimental group volunteers underwent a second blood collection 24 h after the first. Glycemia, insulin status and lipid profile were measured and Homa IR and Homa-beta were calculated. The t-test for independent and dependent samples was used, and a level of significance of 5% was adopted.

**Results:**

Physical exercise changed the glycemic response in both the intragroup analysis (before = 96 ± 6.6 mg/dL vs after = 92 ± 6.6 mg/dL), (*p* = 0.01), and in the intergroup analysis (control = Δ 0.9 ± 6.1 vs experimental = Δ -4.1 ± 6.3) (*p* = 0.02). No changes were shown for the Homa IR, Homa Beta and Insulin indexes. When the lipid profiles were evaluated, differences in HDL were shown in the intragroup analysis (before = 89 ± 10.5 mg/dL vs. after = 91 ± 10.3 mg/dL) (*p* = 0.04). For the other parameters (LDL, TG, Total Cholesterol, TG/HDL), no changes were shown.

**Conclusion:**

In women with excess body weight, a low intensity exercise session diminished the glycemia, but did not change the lipid response.

**Trial registration:**

NCT03170973. Retrospectively registered.

## What does this study add?

- No clinical trial evaluating the acute effect of physical exercise based on energy expenditure on the metabolism of women with altered body weight was identified.
- The results on the effect of acute exercise on metabolism have not yet been fully elucidated with divergent conclusions.

## Background

Various studies have evaluated the acute and chronic effects of physical exercise on metabolic variables [1, 2]. The large majority of these studies have observed that physical exercise was an efficient therapy regulating both the lipid and glycemic profiles.

More specifically, the effects of a single session of physical exercise have also been the object of study of some researches [3–5]. Along this line, there is a strong counter point between the studies, since some have shown positive effects, while other pointed out no changes in the lipid or glycemic profiles with only one session [4, 5].However, all the studies that have evaluated the effect of a single exercise session on the metabolic response, raised the hypothesis that there are various factors that influence the results, such as: the clinical condition and previous functional capacity of the participants, and the characteristics of the exercise applied. Moreover, it is known that one of the clinical conditions with great influence on this response is the Body Mass Index (BMI). Furthermore, it has been demonstrated that obese persons with dyslipidemia or diabetes mellitus present more positive results when compared with eutrophic populations [6, 7]. Caloric expenditure has been pointed out as being the main variable of physical exercise that determines the beneficial effect on the lipid profile [8]. Thus, the aim of this study was to test the hypothesis that a physical exercise session based on caloric expenditure may acutely modify the variables of glycemia and lipid values of women with excess body mass index.

## Methods

### Study design and population

This was a Randomized Clinical Trial registered in the Clinical Trial Registry with the identifier number NCT03170973. The accessible population came from the Clinic School of the “Faculdade Adventista da Bahia”, in Cachoeira, BA, Brazil.

All the women registered with the Physical Therapy Service of the Clinic School who had a body mass index (BMI) above 24.9 kg/m^2^ were invited to participate in the study. A total of 66 volunteers fulfilled the inclusion criteria, which were: age between 18 to 30 years, BMI > 24.9 kg/m^2^ and be sedentary. Sedentarism was determined based on the International Physical Activity Questionnaire - long version [9].

Excluded from the study were women who presented cardiovascular; metabolic disease; hypothyroidism; renal parenchymal disease or diabetes mellitus; history of alcoholism or smoking; use of hypolipemiant, corticosteroid, diuretic, beta-blocker, and contraceptive medications.

The women were randomly divided into two groups: exercise and control; both with 33 volunteers.

### Exercise group

After 12 h fasting, the volunteers were submitted to blood collection, in the antecubital vein, to measure the basal serum values of triglycerides, total and fractionated cholesterol, glycemia and insulin. From the Glycemia and Insulin values, the Homa-IR and Homa-Beta index values were calculated by means of the equation proposed by Mathews and cols [10]. The patients were evaluated relative to diet on the two days before the blood exam, by means of a 24-h food intake diary, with a view to minimizing the effects of diet on the results.

When 12 h had elapsed after the first blood collection, the patients performed a physical exercise session on an ergometric treadmill. The exercise session was divided into 3 time intervals: warming-up, conditioning and cooling-off. The duration of warming-up was 7 min; cooling off 5 min, and conditioning time corresponded to energy expenditure of 250Kcal [11]with light intensity based on the Borg [12]. Rating of Perceived Exertion (RPE), that is, on the original scale - a value between 9 and 11. For better understanding of this scale, on the day before the exercise, the volunteers were familiarized with the RPE concept to allow them to get used to answering in an adequate manner when they were asked about the intensity of the exercise. A cardiac frequency meter was used, which measured the energy expenditure based on the volunteer’s body mass, sex and age.

After the physical exercise session, they were instructed to go home and keep to their habitual diet. When 24 h had elapsed after the first blood collection, the volunteers returned to the laboratory after a 12-h fast, and once again had blood samples collected. Once again, they were questioned about their diet on the day before the exam by means of keeping a 24-h food-intake diary.

### Control group

**Fig. 1:**
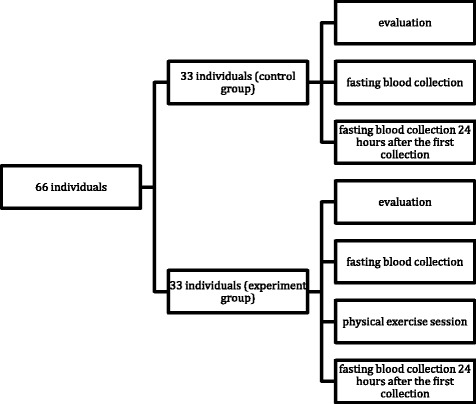
Flowchart of data collection

The women in the control group were submitted to the same data collection protocol as that of the experimental group, however, they did not do the exercise 12 h after the first blood collection. They were instructed not to perform any physical exercise on the two days before the blood collection, as shown in the flow diagram presented below. (Fig. 1).

### Blood collection and metabolic profile

The volunteers were submitted to blood collection after fasting for 12 h. Samples of 5 ml of blood were collected in tubes with EDTA, and after collection the samples were centrifuged at a speed of 3000 rpm for 10 min.

Serum analyses were performed as follows: the blood glucose levels, triglycerides and total cholesterol were determined by the enzymatic calorimetric method; LDL was calculated by using the Friedwald [13] equation; and the Homa-IR and Homa-Beta indexes were calculated by using the equation proposed by Mathews and cols [10].

### Ethical aspects

This study was submitted to the Research Ethics Committee of the Faculdade Adventista da Bahia and approved under Protocol No. 34017514.5.0000.0042. Throughout the entire study, the guidelines on research with human beings of Resolution 466/2012 of the National Health Council were observed.

### Statistical analysis

The data were previously analyzed by the Shapiro-Wilk test, with regard to symmetry. For characterization of the following variables: BMI, age, HOMA-IR, HOMA–beta, Insulin, glycemia, TG, CT, HDL, LDL and TG/HDL, the mean and standard deviation or median and interquartile interval were used, depending on the behavior of the variable. The level of significance was defined by the value of *p* < 0.05.

For comparison of the effects of exercise on the glycemic, lipid and inflammatory profiles, inter- and intragroup comparisons were made by using the paired and non-paired Student’s-*t* test in cases of symmetry, and the Mann–Whitney and Wilcoxon Signed-Rank tests in cases of non-parametric data. The data were analyzed by using the Statistical Package for the Social Sciences (SPSS) software program, version 14.0.

## Results

This study included 66 young women, aged 24 ± 3.6 years, with BMI 29 ± 4.3Kg/m^2^, with lipid and glycemic profiles within the values of normality.. Metabolic values ​​do not differ between the experimental and control groups except for the insulin and Homa values ​​that are higher in the control group. The clinical characteristics are described in Table 1.

**Table 1:** Clinical and anthropometric characteristics of the total sample and per group on the first day of blood collection

VARIABLES	Total Sample (*n* = 66)	CG (*n* = 33)	EG: (*n* = 33)	*p*
Triglycerides (mg/dL)	94 ± 43	99 ± 43	102 ± 64	0.81
Total Cholesterol (mg/dL)	162 ± 32	163 ± 29	159 ± 30	0.59
High Density Lipoprotein (mg/dL)	49 ± 10	45 ± 8	49 ± 10	0.11
Low Density Lipoprotein (mg/dL)	94 ± 28	97 ± 24	89 ± 26	0.18
Glycemia (mg/dL)	84 ± 8	84 ± 9	82 ± 8	0.22
Insulin (mcIU/mL)	10 ± 5	12 ± 6	8 ± 5	0.01*
Homa IR	2.4 ± 1.2	2.9 ± 1.4	2.0 ± 1.3	0.01*
Homa-Beta	34 ± 20	40 ± 19	27 ± 18	0.01*

CG*-*Control Group; EG-Experimental Group; *p<0.05, Student's-*t* Test

In the intragroup analysis, a decrease in serum glycemia (96.7 ± 6.6 vs 92.6 ± 6.6 mg/dl) (*p* = 0.01) was observed in experimental group, only. No difference was demonstrated for the others variables in both groups (Table 2).

**Table 2:** Intragroup analysis of glycemic values, insulin and HOMA IR and HOMAbeta

	Before	After	*P*
EG (*n* = 33)			
Glucose (mg/dL)	97 ± 6.6	93 ± 6.6	0.01*
Insulin (mcIU/mL)	8 ± 5.2	8 ± 5.2	0.99
Homa Index	2.0 ± 1.3	1.9 ± 0.9	0.69
Homa-Beta	27.9 ± 18.7	27.6 ± 18.7	
CG (*n* = 33)			
Glucose (mg/dL)	97 ± 8.6	98 ± 8.8	0.41
Insulin (mcIU/mL)	12 ± 5.6	12 ± 5.2	0.78
Homa IR	2.9 ± 1.4	2.8 ± 1.3	0.76
Homa-Beta	40.7919.7	39.4 ± 17.1	0.70

EG = Experimental Group; CG = Control Group; *Bidirectional Student’s*-t* Test for paired samples

When the intergroup glycemic profile variation was analyzed, the decrease of glucose was lower in the experimental group. No difference was observed in insulin level, insulin resistance and insulin sensitivity between groups. (Table 3).

**Table 3:** Comparison of variation in the glycemic profile in the Control and Exercise Groups

	CG (*n* = 33)	EG (*n* = 33)	*p*
Δ Glucose	0.90 ± 6.1*	−4.18 ± 6.3	0.02*
Δ Homa-IR	−0.06 ± 1.2*	−0.06 ± 0.9	0.99
Δ Homa-Beta	−1.30 ± 19*	−0.30 ± 13.8	0.81
Δ Insulin	0.00 ± 5.3*	−0.18 ± 3.8	0.87

EG = Experimental Group; CG = Control Group; *Bidirectional Student’s*-t* Test for independent samples

The intra-group analysis of lipid profile, showed a significant increase in the HDL values and a tendency to decrease in the TG/HDL ratio in the exercise group, while in the control group, no change was found in any of the lipid profile variables (Table 4).

**Table 4:** Intragroup Lipid Profile Analysis (*n* = 33)

	Before	After	*p*
Experimental			
Total Cholesterol (mg/dL)	159 ± 30.3	161 ± 34.0	0.36
Triglycerides (mg/dl)	102 ± 64.4	93 ± 49.3	0.08
High Density Lipoprotein (mg/dL)	49 ± 10.5	51 ± 10.3	0.04
Low Density Lipoprotein (mg/dL)	89 ± 26.4	91 ± 29.7	0.27
Ratio TG/HDL	2.2 ± 1.6	1.9 ± 1.2	0.06
Control			
Total Cholesterol (mg/dL)	163 ± 29.0	162 ± 30.9	0.77
Triglycerides (mg/dl)	99 ± 42.6	94 ± 37.4	0.06
High Density Lipoprotein (mg/dL)	46 ± 7.7	46 ± 10.3	0.09
Low Density Lipoprotein (mg/dL)	97 ± 23.6	97 ± 26.8	0.69
Ratio TG/HDL	2.2 ± 1.1	2.1 ± 1.1	0.10

Student’s*-t* test for dependent samples

In the intergroup comparison, no difference was observed in the variation of triglycerides, total cholesterol, LDL-C, HDL-c and TG/HDL levels, neither in the experimental group nor in the control group (Table 5).

**Table 5:** Intergroup Analysis of Variation in Lipid Profile

	Control	Experimental	*p*
Δ Total Cholesterol	−1.0 (−6.5–3.5)	0.0 (−0.4–6.5)	0.32
Δ Triglycerides	−4.0 (−13.0–4.5)	−5.0 (−19.0–11.0)	0.80
Δ HDL	0.0 (−1.5–4.1)	0.6 (−1.0–2.8)	0.80
Δ LDL	−3.0 (−6.5–3.5)	0.0 (−4.0–6.5)	0.62
Δ TG/HDL	−0.1 (−0.3–0.1)	−0.1 (−0.4–0.1)	0.32

Median (Interquartile Interval); Mann–Whitney Test

## Dsiscussion

The results of this study demonstrated that low intensity physical exercises in women with excess weight, acutely reduced the serum glycemia, however, it did not change the lipid profile.

Some studies, conducted with other populations and different protocols have corroborated our results and pointed out that/?this type of?/exercise was incapable of improving the lipid profile in an acute manner [4]. However, in the study conducted by Ferguson et al. [14], the correspondence was investigated, between the energetic threshold and the possible changes in the triglyceride levels and concentrations of lipoproteins in trained men after four exercise protocols. The protocols were carried out with caloric expenditures of 800, 1100, 1300 and 1500 kcal. Twenty-four hours after performing the sessions, the HDL was significantly elevated in the exercises with expenditure of 1100, 1300 and 1500 kcal. Whereas the LDL concentration diminished significantly with an expenditure of 1300 kcal; and that of triglycerides, with 800 kcal after one single exercise session. In the same study, it was possible to observe an increase in lipoprotein lipase activity 24 h after the sessions with caloric expenditure of over 1100 kcal, and this remained elevated up to 48 h after the session using 1500 kcal, as these changes coincided with the changes in HDL. In another study, Ferreira et al. [15] also observed significant reduction in post-prandial lipemia in men submitted to different intensities of effort both with caloric expenditure of 500 kcal. They verified that both moderate and high intensity exercise presented reduction in post-prandial lipemia. Possibly the caloric expenditure on performing the protocol of this study was not enough to promote these changes.

Nevertheless, the protocol used was effective in reducing glycemia. The knowledge that exercise increases insulin sensitivity,, in both the acute and chronic form, served as a basis for explaining the results obtained in this study [16].

Some are the effects promoted by exercise, which explain this result. Physical exercise is known to increase the phosphorylation of insulin receptors (IRS1 and 2), which consequently facilitates the action of insulin [17]. This effect occurs during exercise and may last of up to 16 h after the exercise [18].

More specifically, in obesity, changes occur in diverse points of the insulin signal transduction pathway. Such as reduction in the concentration and phosphorylation of the insulin receptors [19]. In many cases, this is explained by the higher level of subclinical inflammation in this population [20]. Hypertrophy of the adipose tissue stimulates the production of prof-inflammatory adipokines such as TNF-alfa and diminishes the production of anti-inflammatory substances such as adiponectin. This may consequently diminish insulin sensitivity, since TNF-alfa hinders, and adiponectin favors the action of insulin [21]. On the other hand, this process of physical exercise attenuates the sub-clinical inflammation, and improves the relations between the production of pro- and anti-inflammatory substances by the adipose tissue [9]. Although studies with only one exercise session have presented controversial results in this population [22, 23], thus acute reduction in subclinical inflammation is also a possible mechanism that explains the reduction in glycemia in the EG.

Other mechanisms independent of insulin may also explain the reduction in glycemia in the EG. The increase in bioavailability of chrome that occurs during and after exercise appears as one of the explanations. During exercise the increase in the need of glucose in muscle tissue stimulates the release of chrome that acts as adjuvant to insulin. Chrome potentiates the action of insulin, stimulating glucose absorption during and after exercise, increasing the fluidity of the cell membrane to facilitate insulin binding to its receptor [24]. This increase in the blood concentration of chrome may last for hours after exercise. The higher level of calcium release by the sarcoplasmic reticulum also favors glucose transport to the muscle cell [25]. The increase in calcium in the muscle cell cytoplasm initiates and facilitates activation of the molecules involved in the intracellular signaling cascade of glucose transport [25].

A mechanism that is also independent of insulin is that of the AMPK enzyme (AMP-activated protein kinase). This enzyme stimulates glucose transport in the skeletal muscle. Its activation results in a reduction in the stocks of intracellular glucose. In the situation in which the AMP:ATP ratio increases, an increase in AMPK activity also occurs. This increase in AMPK activity in response to a need of generating ATP, particularly during but also after physical exercise, promotes the translocation of vessels containing Glut-4 [26]. This finally facilitates the influx of glucose into the muscle cell independently of insulin.

Lastly, another possibility may be associated with exercise-induced changes in hemodynamics. A single session of exercise is known to diminish sympathetic activity and increase muscle blood flow in the period after exercise. It is interesting to note that after a single session of exercise, sympathetic action diminishes and muscle vasodilatation increases. These and other hemodynamic changes may also contribute to increasing insulin sensitivity after exercise [27]. Moreover, physical exercise stimulated the production of endothelial nitric oxide synthase (eNOS), by means of shear stress on endothelial cells during exercise [28]. At the same time it inhibits a series of molecules that favor the production of Inducible nitric oxide synthase (iNOS). While the former is related to the higher level of vasodilatation of the active musculature [29], Inducible nitric oxide synthase is associated with insulin resistance [30]. Studies with rats have pointed out that a single session was capable of promoting a reduction in this enzyme and consequently increase insulin sensitivity.

Some of points of this study should be emphasized. The authors observed that although the study population was randomly divided, the CG presented higher insulin, Homa-IR and Homa-Beta values than the EG. Could this have influenced the results obtained? Individuals with a lower level of insulin sensitivity are known to present a higher concentration of plasma insulin in an attempt to maintain an adequate supply of glucose within the muscle and adipose cells. This was perceived when the HOMA-Beta values rose, demonstrating a higher level of insulin production by the beta-pancreatic cells.

Individuals who presented this condition had greater difficulty with metabolizing lipids, because when insulin binds to its muscle membrane receptors, it stimulates the action of lipoprotein lipases that play a fundamental role in the metabolism of triglycerides and plasma lipoproteins. However, as observed in Table 1, the fasting lipid profile values did not differ between the groups. Whereas, as observed in Table 4, in the EG there was a significant reduction in LDL and the Ratio TG/HDL, while no reduction was observed in the CG. Nevertheless, this reduction was not sufficient to show difference in the intergroup comparison.

Among the limitations of this study, the authors point out a single 12-h time interval of observation, and absence of caloric expenditure above 250Kcal for different comparisons. With regard to physical activity, it is known that factors such as time of observation and energy expenditure may influence the plasma limit response [31]. The authors observed that this study signals the responses to a caloric expenditure and in a specific time, and in this case no change was identified in the lipid response; which does not mean that these responses did not occur in later time intervals or in higher caloric expenditures. Comparisons of different protocols in the acute and sub-acute stage of physical exercise must be tested so that lipid responses to different energy expenditures and in different time intervals may be shown after the physical exercise session.

## Conclusion

In overweight women, the sub-acute effect of low intensity physical exercise is capable of modifying the glycemic levels, not interfering in the lipid response and insulin resistance variables.
